# Abnormal Frontostriatal Activity During Unexpected Reward Receipt in Depression and Schizophrenia: Relationship to Anhedonia

**DOI:** 10.1038/npp.2015.370

**Published:** 2016-01-20

**Authors:** Nuria Segarra, Antonio Metastasio, Hisham Ziauddeen, Jennifer Spencer, Niels R Reinders, Robert B Dudas, Gonzalo Arrondo, Trevor W Robbins, Luke Clark, Paul C Fletcher, Graham K Murray

**Affiliations:** 1 Department of Psychiatry, University of Cambridge, Cambridge, UK; 2 Wellcome Trust-MRC Institute of Metabolic Science, Cambridge, UK; 3 Cambridgeshire and Peterborough NHS Foundation Trust, Cambridge, UK; 4 Liaison Psychiatry Service, Norfolk and Suffolk NHS Foundation Trust, Ipswich, UK; 5 Department of Psychology, University of Cambridge, Cambridge, UK; 6 Institute of Behavioural and Clinical Neuroscience, University of Cambridge, Cambridge, UK; 7 Department of Psychology, Centre for Gambling Research at UBC, University of British Columbia, Vancouver, Canada

## Abstract

Alterations in reward processes may underlie motivational and anhedonic symptoms in depression and schizophrenia. However it remains unclear whether these alterations are disorder-specific or shared, and whether they clearly relate to symptom generation or not. We studied brain responses to unexpected rewards during a simulated slot-machine game in 24 patients with depression, 21 patients with schizophrenia, and 21 healthy controls using functional magnetic resonance imaging. We investigated relationships between brain activation, task-related motivation, and questionnaire rated anhedonia. There was reduced activation in the orbitofrontal cortex, ventral striatum, inferior temporal gyrus, and occipital cortex in both depression and schizophrenia in comparison with healthy participants during receipt of unexpected reward. In the medial prefrontal cortex both patient groups showed reduced activation, with activation significantly more abnormal in schizophrenia than depression. Anterior cingulate and medial frontal cortical activation predicted task-related motivation, which in turn predicted anhedonia severity in schizophrenia. Our findings provide evidence for overlapping hypofunction in ventral striatal and orbitofrontal regions in depression and schizophrenia during unexpected reward receipt, and for a relationship between unexpected reward processing in the medial prefrontal cortex and the generation of motivational states.

## Introduction

Depression and schizophrenia are associated with deficits in motivation and enjoyment, which have been collectively termed anhedonia by some authors (James, 1902). Clinically it is challenging to distinguish between deficits in pleasure and motivation, even though there are at least partially separable processes that underpin these functions (Berridge and Robinson, 2003). Some evidence suggests that striatal hypofunction during reward processing is present in schizophrenia and depression, and may contribute to the motivational and hedonic problems experienced by patients (Juckel *et al*, 2006; Smoski *et al*, 2009), whilst other authors have emphasized the importance of orbitofrontal cortex function in this regard (Morris *et al*, 2011; Gold *et al*, 2012). It remains unknown whether the neurobiological disturbances in reward processing are different in schizophrenia and depression and it is not yet clear which aspects of reward processing are particularly problematic in these conditions, or whether any associated neural deficits are predominantly cortical or subcortical in origin. Insights into any shared pathophysiology underlying abnormalities in motivation and pleasure across different psychiatric diagnoses could lead to improved use of existing treatments, facilitate development of new treatments and, as is postulated by the Research Domain Criteria (RDoC) project, may contribute to improved psychiatric classification in the future (Heinz *et al*, 1994; Insel *et al*, 2010; Hägele *et al*, 2014).

Recently it has been suggested that in both schizophrenia and depression, the aspects of reward processing that relate to reward receipt may be relatively spared, whereas anticipatory and motivational aspects of reward processing may be more dysfunctional and may be closely linked to negative symptoms/depression (Argyropoulos and Nutt, 2013; Dillon *et al*, 2014; Kring and Caponigro, 2010; Barch and Dowd, 2010). However, the majority of neuroimaging studies that have examined reward receipt in schizophrenia and depression have used tasks where rewards are rather predictable, and can be expected to occur more often than not; several studies using such tasks have found broadly intact neural responses to reward feedback in schizophrenia and depression (eg, Abler *et al*, 2008; Simon *et al*, 2010; Smoski *et al*, 2011; Dowd and Barch, 2012; Gilleen *et al*, 2015). Such tasks are optimized to examine brain responses during the anticipation and receipt of a highly expected reward, rather than unexpected reward responses; several studies have documented robust striatal and cortical deficits in schizophrenia and depression in the anticipation of reward.

This still leaves open the question as to whether response to reward receipt, especially unexpected reward receipt, is normal in these disorders (Barch and Dowd, 2010; Strauss *et al*, 2014). In this regard it is critical to examine neural responses to unpredicted rewards, which may be more pronounced than those to predicted rewards (Schultz *et al*, 1997). This is of particular interest, given that unexpected events evoke prediction errors, which are encoded within the brain at both cortical and subcortical levels (Schultz and Dickinson, 2000; Garrison *et al*, 2013). Prediction error signaling has been postulated to relate to many aspects of thought and behavior in health and in psychiatric illness, including learning, motivation, and attention, and abnormal brain prediction error signaling may contribute to psychotic symptoms as well as deficits in motivation and enjoyment (Fletcher and Frith, 2009; Murray, 2009; Ziauddeen and Murray, 2010; Gradin *et al*, 2011).

Initial evidence suggests that the prediction error signaling during, or after, learning may be compromised in both schizophrenia and depression in cortical and subcortical regions (Murray *et al*, 2008; Kumar *et al*, 2008; Waltz *et al*, 2009; Gradin *et al*, 2011; although see Dowd and Barch, 2012). However, abnormal neural correlates of prediction error associated learning signals may reflect dysfunction of the learning mechanism (eg, failure to update in response to prediction error signals during learning), not necessarily to the prediction error signaling mechanism *per se*. To fully assess the integrity of neural systems that signal surprising/unexpected rewards, it is critical to employ an experimental scenario with little or no learning component in which reward outcome is unpredictable (Morris *et al*, 2012).

Our aim therefore was to explore brain responses to unexpected reward delivery in both depression and schizophrenia, and their relationship to motivation and enjoyment. We used an fMRI reward processing task involving the receipt of unexpected rewards, but minimal learning, with a sample of patients who all subjectively endorsed at least some degree of anhedonia. Our goals were:

1. to test whether brain responses to unexpected rewards were broadly intact in schizophrenia and depression,
2. if brain responses to unexpected rewards are abnormal in schizophrenia and depression, to evaluate if the deficits are confined to cortical, or subcortical regions, and whether there are any shared or differential areas of deficit in the two disorders, and
3. to examine whether brain responses to unexpected reward receipt are related to motivation and enjoyment in these disorders.

## Materials and methods

### Participants

The study was approved by the Cambridgeshire 3 National Health Service research ethics committee. Written informed consent was obtained from all participants.

Twenty-one people with DSM-IV schizophrenia, 24 people with DSM-IV major depressive disorder (MDD), and 21 healthy volunteers took part in the study (Table 1). All schizophrenia participants were taking antipsychotic medication; eight were additionally taking antidepressant medication. Thirteen of the 24 depression participants were taking antidepressant medication, of whom four were additionally taking antipsychotic medication; medication is described in Table 1 and in further detail in Supplementary Material. Inclusion criteria were an age between 18 and 65 and adequate proficiency in English. Exclusion criteria were history of neurological disorder, physical illness, dependence on alcohol or recreational drugs, and any contraindication for MRI scanning. A first-degree family history of schizophrenia or bipolar disorder was an additional exclusion criterion in the depression and control groups. All participants with depression or schizophrenia subjectively endorsed a degree of loss of interest or pleasure.

### Anhedonia Assessment

To assess anhedonia we used the Snaith Hamilton Pleasure Scale (SHAPS), a validated self-report measure (Snaith *et al*, 1995; Franken *et al*, 2007).

### fMRI Task Description

**Figure 1:**
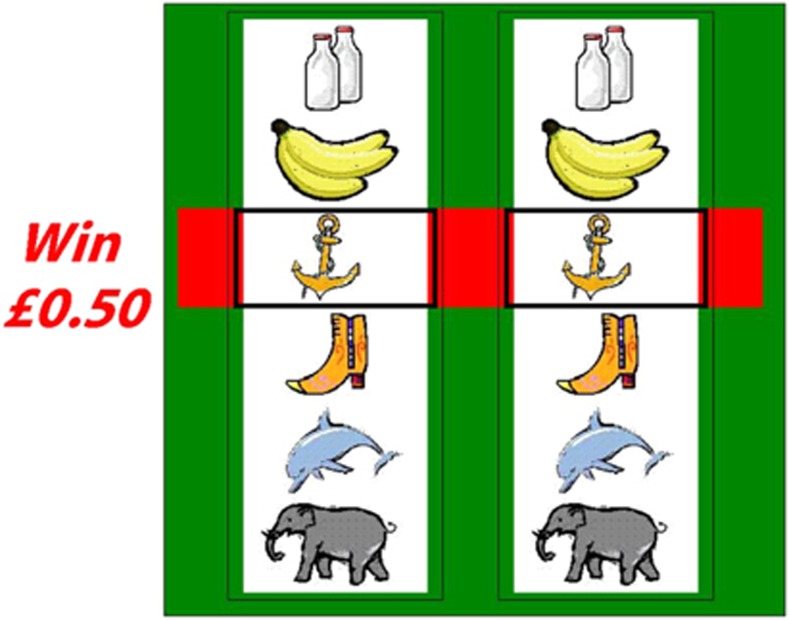
Example of a ‘win trial' in the fMRI simulated slot-machine game.

The task involved playing a computerized version of a slot-machine game; participants view two reels of a slot-machine/one arm bandit game, where the left hand reel is stationary and the right hand reel spins until it stops (Figure 1). If the two icons in the center of view match, there is a financial reward of 50 pence. Participants win on an average one in six trials, making rewards unexpected in this game. Furthermore, the duration of the spinning of the wheel is variable in this task (delay varies between 2.8 and 6 s), so the precise timing of the outcome is also not predictable. The game consisted of two runs of 60 trials, each run lasting ~20 min, and has been previously described (Clark *et al*, 2009). On 50% of trials, the participant selects the ‘play icon'—the image in the center of the left hand reel—by rotating the reel to the icon of their choice. In the other 50% of trials (pseudorandomised distribution), the computer selects the ‘play icon' on these trials the participant is required to confirm with a button press that he/she has noted the computer choice. After this selection phase the right hand reel starts to spin. The selection phase lasts 5 s, followed by a variable delay stage whilst the second reel spins and comes to a stop, followed by an outcome phase of 4 s where the reward is presented: ‘£0.50 win!' (if the icons on the payline of the two reels match) or ‘No win' (if they do not match). If selection/confirmation did not occur within 5 s, ‘too late' was presented on the screen and the task moved on to the next trial. At the end of each trial, there was a variable inter-trial interval of between 2 and 7 s duration. Participants were told that they would be given any money they won at the end of the experiment.

#### Task-related pleasure and motivation

Immediately after the scan session the participants answered the questions, ‘When the second picture matched the chosen picture you won money. How much did you like the feeling of winning money?' and, ‘When the second picture matched the chosen picture you won money. Did this make you want to play more?' The answers were marked on a visual analog scale.

### fMRI Data Acquisition and Pre-Processing

A Siemens Trio Tim operating at 3T was used to collect imaging data. Gradient-echo T2*-weighted echo planar images depicting BOLD contrast were acquired from 32 noncontiguous oblique axial planes to minimize signal drop-out in ventral regions. TR=2 s; echo time=30 ms; flip angle=78; voxel size=3.14 × 3.14 × 3.75 mm^3^, matrix size 64 × 64; bandwidth 2232 HZ/Px. A high-resolution T1-weighted three-dimensional MP-RAGE structural image was also acquired for use in spatial normalization of the EPI series. Imaging data was analyzed using FSL software (FMRIB's Software Library, www.fmrib.ox.ac.uk/fsl). See Supplementary Material for pre-processing details.

### fMRI Data Analysis

An event-related analysis in FSL software was used to identify neural responses at the time of the unexpected win. We used a single statistical linear regression model with four explanatory variables and their temporal derivatives: (a) anticipation phase (the duration of this event varied between 2.8 and 6 s on different trials); (b) win outcome (4 s duration, 20 events in total); (c) near-miss outcomes (4 s duration, 40 events in total); (d) full-miss outcomes (4 s duration, 60 events in total). A near-miss outcome is where the play icon finishes adjacent to, but not on, the payline; near-miss outcomes have been shown to evoke neural responses different to other misses (Clark *et al*, 2009). Movement parameters from the realignment step were also included in the first-level model.

As our hypotheses concerned brain activation in response to unexpected reward, an ‘unexpected reward receipt' contrast was investigated, formed by the contrast of win outcomes *vs* full-miss outcomes. This contrast was computed at the single-participant level and the *β*-parameters for participants from this contrast were carried forward to group analyses. One-way between participants ANOVA was conducted at the whole-brain level to compare between the three groups. The one-way ANOVA only identifies regions in which activation is different between groups, without indicating which groups drive the differences or the directionality of the differences. Therefore, for clusters in which the ANOVA indicated group differences we extracted the mean parameter estimates for each subject for that cluster (using the FSL tool Featquery) and conducted *post hoc* comparisons across groups. Regression analyses at the whole-brain level in FSL were used to investigate relationships between brain activation and the post-scan subjective ratings of motivation and pleasure.

Imaging comparisons were cluster thresholded using the FSL tool easythresh, using a family-wise error (FWE) correction at *p*<0.05 (initial cluster threshold *z*=2).

## Results

### Demographics and Rating Scales

Groups did not differ in age, gender, handedness, years of education, IQ, or ethnicity (Table 1).

Patients with depression had higher SHAPS anhedonia scores than healthy controls and patients with schizophrenia. Patients with schizophrenia had higher SHAPS anhedonia scores compared with controls (Table 1).

The three groups did not differ on a visual analog measure of how much they valued 50 pence (answer to question ‘how often do you pick up a 50 pence coin when you see it in the street' *p*=0.843).

### Behavioral Analysis

#### Reward task wins

There were no differences in the number of wins according to group, reflecting the pseudo-randomized nature of the paradigm (wins F=2.23, df=2,65, *p*=0.11).

#### Reward task post-scan questions (reward liking and task motivation)

Participants with depression reported significantly lower liking of the feeling of winning money than controls (depression: mean=56.3, SD=23.2; controls: mean=74.3, SD=21.0; t=2.7, df=43, *p*=0.009). In this measure of liking there was a marginal difference between schizophrenia participants (mean=61.1, SD=27.9) and controls (*t*=1.75, df=40, *p*=0.09), and no difference between schizophrenia and depression participants (*t*=0.65, df=43, *p*=0.52).

In response to the question ‘When the second picture matched the chosen picture you won money. Did this make you want to play more?', there was a significantly higher score in controls (mean=67.0, SD=29.2) than depression patients (mean=44.4, SD=25.7; comparison *t*=2.87, df=42, *p*=0.009). Controls scored higher than schizophrenia patients on this measure with marginal statistical significance (schizophrenia: mean=48.3, SD=30.4; comparison controls *vs* schizophrenia *t*=2.0, df=39, *p*=0.05). There was no significant difference between depression and schizophrenia on this motivational measure (*t*=0.66, df=43, *p*=0.64). One control had missing data on this measure.

*Associations with anhedonia questionnaire.* Higher SHAPS anhedonia scores were associated with lower reported motivation ratings in the whole sample (*β*=−0.3, *t*=2.6, *p*=0.01), and in the schizophrenia group (*β*=−0.7, *t*=4.3, *p*<0.001). SHAPS anhedonia also negatively predicted the pleasure ratings after a win in the whole sample (*β*=−0.3, *t*=−2.2, *p*=0.03).

### Brain Imaging Results

We evaluated activation associated with unexpected reward receipt using the contrast of a win outcome *vs* full-loss outcome.

#### Entire sample (one sample t-test)

**Figure 2:**
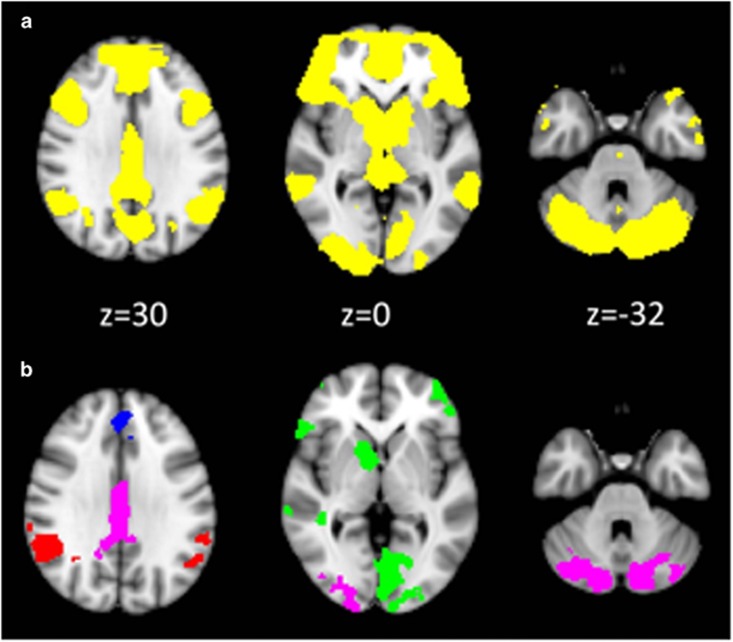
fMRI results: receipt of unexpected reward. Left hemisphere is shown in the right side of the image. Coordinates are expressed in mm, and in standard space. Panel a: entire sample pooled analysis. The yellow color indicates significant voxels thresholded at *p*<0.05 family-wise error (FWE) voxelwise corrected for illustrative purposes. Panel b: between groups analysis using ANOVA. Colored clusters indicate those clusters indicating significant group differences (cluster threshold *Z*>2.0 *p*<0.05 FWE whole-brain cluster corrected) and the particular color indicates the results of *post hoc* tests. Green areas are clusters where the control group has greater activation compared with the depression group and compared with the schizophrenia group; blue areas are clusters where controls have greater activation compared with the depression group and compared with the schizophrenia group, and the depression group has greater activation than the schizophrenia group; red areas are clusters where controls have more activation than the schizophrenia group and the depression group has more activation than the schizophrenia group; lilac areas are clusters those where the control group has more activation than the schizophrenia group.

There was a highly significant brain response in a widespread cortico-striatal network including the striatum, orbitofrontal cortex and medial prefrontal cortex. The maximum significance was located in left caudate (MNI *x*=−8, *y*=10, *z*=2; *Z*-score=9.28; Figure 2, Supplementary Figures S1 and S4).

#### Between group analysis

**Figure 3:**
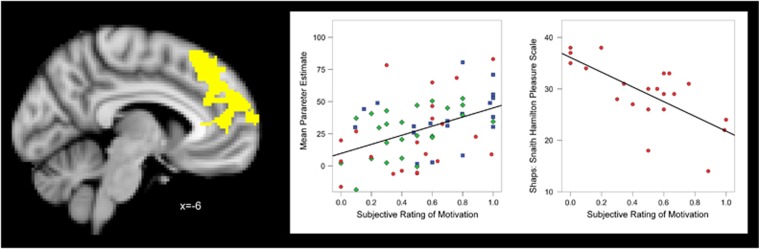
Left panel: yellow color indicates a significant cluster resulting from the linear regression of brain activation during an unexpected win against subjective ratings of task-related motivation (all participants pooled, adjusted by group). Left hemisphere is shown in the right side of the image. Coordinates are expressed in mm, and in standard space. FWE whole-brain corrected, cluster threshold *Z*>2.0 *p*<0.05. Centre panel: scatterplot of the extracted contrast parameter estimates during an unexpected win from the cluster depicted in left panel *vs* subjective task-related motivation (blue square, controls; green triangle, depression; red circle, schizophrenia). Right panel: scatterplot of SHAPS (Snaith Hamilton pleasure scale) score *vs* the subjective rating of motivation in the schizophrenia group.

One-way ANOVA analysis demonstrated 10 clusters with significant group differences (Figure 2, Table 2, Supplementary Figures S2). *Post hoc* pairwise tests of mean cluster parameter estimates showed that, in comparison to healthy controls, MDD and schizophrenia participants had a decreased signal (eg, Figure 3 upper panel) to unexpected rewards in clusters that encompassed bilateral orbitofrontal cortex, right caudate, right nucleus accumbens, right midbrain, right thalamus, right inferior and middle temporal gyrus, and left occipital cortex.

The *post hoc* tests also revealed that in the medial prefrontal cortex, in a cluster including the paracingulate gyrus and superior frontal gyrus, both patient groups had reduced activity compared with controls, and the schizophrenia group also had significantly lower activation compared with depression.

Compared with controls, the schizophrenia group had significantly lower activations in the posterior division of the cingulate gyrus, right occipital pole, and bilateral cerebellar areas.

The posterior lateral parietal cortex bilaterally (angular and supramarginal gyri, and intraparietal sulci) showed reduced activation in schizophrenia (compared with depression and controls) but there were no differences here between depression and controls (Figure 3, lower panel).

Our results are based on the contrast of unexpected reward receipt *vs* full miss. Examination of the parameter estimates for the regressors that make up this contrast (ie, their activation compared with baseline) suggest that the group differences were secondary to reduced patient responses to unexpected reward receipt rather than differences in full-miss activation (Supplementary Figure S25).

#### Associations with subjective experience

**Figure 4:**
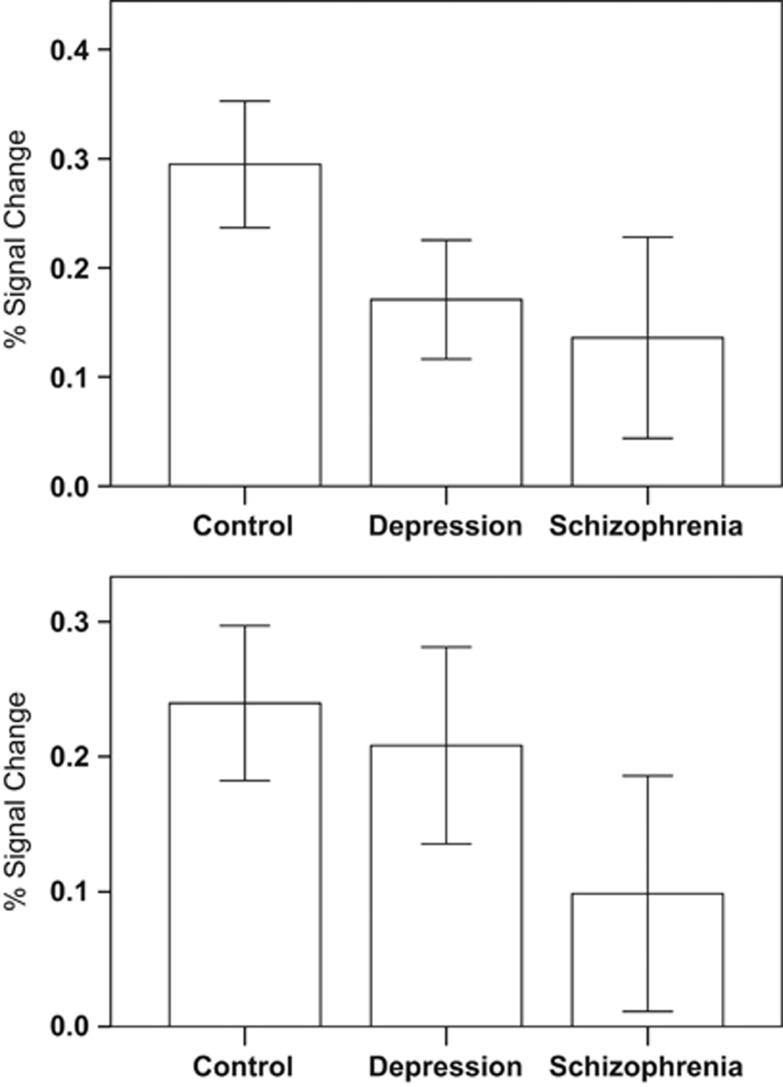
The upper panel shows the mean percent signal change for unexpected reward receipt for the left ventral striatal/orbitofrontal cluster where both patient groups had suppressed activation relative to controls. In contrast, the lower panel shows the mean percent signal change for unexpected reward receipt in the left lateral parietal lobe (angular gyrus and supramarginal gyrus) where activation was suppressed in schizophrenia compared with controls but relatively intact in depression. Error bars are 95% confidence intervals. For graphs of results of other clusters please see Supplementary Material.

In a regression analysis in which we pooled all participants and, importantly, adjusted for group effects by modeling group means, brain activation during receipt of unexpected reward was positively associated (*p*<0.05 corrected) with the post-scan rating of motivation to keep playing the task after a win in a medial prefrontal cluster situated in the anterior cingulate and paracingulate gyrus and bilateral medial superior frontal gyrus (cluster size=2491, *z* maximum peak voxel=4.17, MNI coordinates *x*=−12, *y*=52, *z*=24; Figure 4; Supplementary Figure S3). The strength of this association did not differ between groups (group X motivation interaction term *p*=0.9). Activation in this region was not significantly associated with SHAPS total score. No associations were found between unexpected reward receipt and the hedonic feeling of winning money at *p*<0.05 corrected.

### Relationship to Medication

There were no differences in activation when comparing those depression participants with and without antidepressant treatment. Within the schizophrenia group, we converted treatment doses into chlorpromazine equivalents and found no associations with brain response. See Supplementary Material for more detail.

## Discussion

We used fMRI to examine brain activation to unexpected reward (positive reward prediction error) without learning confounds. The task we used, which had not been employed before in samples with mental illness, elicited activation to unexpected reward receipt in the classic reward network including striatum, orbitofrontal cortex, and medial prefrontal cortex, and yielded insights into relationships between brain activation and subjective experience during the experiment. We demonstrated that brain responses to unexpected rewards are suppressed in a similar way in both schizophrenia and depression in several regions previously implicated in the pathology of both of these disorders, including the orbitofrontal cortex, ventral striatum, thalamus, and insular cortex. However, there were also areas of deficit unique to schizophrenia, such as the posterior lateral parietal cortex. When we examined relationships between brain activation and task-related emotional reports, we found intriguing associations between medial prefrontal unexpected reward receipt responses and motivational state that could help explain aspects of clinical anhedonia.

### Group Differences

Our results indicated reduced activation to the receipt of an unexpected reward in overlapping regions in schizophrenia and depression: the orbitofrontal cortex, ventral striatum, thalamus, and inferior and middle temporal gyri had reduced activation in both disorders. There is a previous report of reduced ventral striatal reward receipt responses in unmedicated depression participants (Robinson *et al*, 2012); in schizophrenia participants lower striatal activity has been described in response to an uncertain primary reward (Waltz *et al*, 2009) and to an unexpected monetary reward (Morris *et al*, 2012). However, some previous studies have documented that processing of unexpected rewards is intact in schizophrenia, and in some circumstances it is not possible to detect case-control differences in psychological and/or brain responses to rewards (Gard *et al*, 2007; Dowd and Barch, 2012). A variety of methodological factors will influence the likelihood of individual studies showing significant groups differences, including sample size, the present symptoms of the patients, the sensitivity of the particular paradigm involved, and whether a motor response is required or not. In the paradigm used in the present study rewards were not predictable, as they occurred at variable times (between 2.8 and 6 s after the reel started to spin) and were uncommon (occurring in one sixth of trials in a pseudo-randomized pattern), and as such these rewards are associated with positive prediction error.

Because not all patients with schizophrenia or depression have difficulties in motivation and enjoyment, patient heterogeneity may also contribute to variability of the results in the existing literature. Our results, in a study where all patients had at least some degree of anhedonia, indicate that striatal, insular, thalamic, temporal cortex, and orbitofrontal cortex processing of unexpected rewards may be abnormal in both schizophrenia and depression, which may impact on hedonic experience, the ability to learn reward-cue relationships and the generation of motivational states.

One previous study compared reward prediction error responses in participants with depression, participants with schizophrenia and controls (Gradin *et al*, 2011); that study scanned participants during learning, and the current study extends these results to demonstrate, in a task where no learning is required, areas of overlapping and differential response associated with reward prediction error in schizophrenia and depression. In addition, whilst Gradin *et al* (2011) examined brain responses correlating with a composite measure of positive and negative prediction error, our study focuses on positive reward prediction error: given that rewards are infrequent in our task, there are no trials in this task where strong expectancies of a win are violated.

Although our results indicate many shared areas of reward processing deficit in schizophrenia and depression, there are also areas of difference. There were schizophrenia specific deficits in unexpected reward receipt activation in the posterior cingulate gyrus, occipital pole, and cerebellum: these areas were not significantly abnormal in depression, although *post hoc* analysis did not demonstrate significant differences in activation between patient groups. We note there were however significant differences between the patient groups in the lateral parietal cortex, in the supramarginal and angular gyri, and intraparietal sulci, which had abnormal activation in schizophrenia but not depression, confirmed in an additional analysis using exclusive masking (see Supplementary Text and Supplementary Figures S11 and S12). There is previous evidence for the role of this part of the parietal cortex in signaling surprising outcomes (Nieuwenhuis *et al*, 2005; Glascher *et al*, 2010), with some evidence that reward processing in this region is dopaminergically mediated (Medic *et al*, 2014); in this regard it is of interest that dysfunction of this region was specific to schizophrenia. Parietal activation associated with the uncertainty of outcomes has previously been shown to be abnormal in schizophrenia (Paulus *et al*, 2003). In the medial prefrontal cortex, including the superior frontal gyrus and anterior paracingulate, activation in both patient groups was significantly abnormal (showing hypoactivation to unexpected reward receipt) with the schizophrenia group showing significantly greater abnormality than the depression group. Medial frontal and anterior cingulate hypoactivation has been reported during the receipt of an expected reward in schizophrenia (Schlagenhauf *et al*, 2009; Waltz *et al*, 2010), and preclinical studies indicate that these cortical regions are critical for motivated behavior (Walton *et al*, 2002, 2003; Rudebeck *et al*, 2006).

### Relationship Between Brain Responses to an Unexpected Reward And Motivational Scores

Motivation to play the game was positively associated with activation to unexpected reward in a cluster situated in medial prefrontal areas, including the anterior cingulate and paracingulate gyrus, in the whole sample (adjusting for diagnosis); this cluster overlapped with the medial prefrontal cluster in which schizophrenia participants showed lower activation than controls and depression, and depression participants showed lower activation than controls, when receiving an unexpected reward. This evidence of an association between brain response to unexpected reward receipt and motivation, rather than pleasure, adds complexity and nuance to models of reward processing that parse pleasure and motivation into separable psychological processes with distinct neural components. However, a full-model should take account of how unexpected reward-related responses influences future behavior through motivation. Although SHAPS scores were associated with task-related measures of motivation, and brain activation in the medial prefrontal cortex was associated with task-related motivation, there was no direct association between brain activation and SHAPS scores, suggesting that medial prefrontal activation may be indirectly associated with anhedonic symptoms via its association with task-related motivation. Our results suggest that medial prefrontal and anterior cingulate activation to unexpected reward receipt may be a mechanism that enables the brain to update the motivational state to reinforce actions that maximize future reward.

Our finding, linking neural response to a reward receipt with an index of motivation, is reminiscent of the results of Waltz *et al* (2009) who found that putamen and gustatory cortex responses to sweet liquid rewards predicted clinical avolition as measured by the SANS. Our findings extend those of Waltz *et al* (2009) to relationships between reward receipt and motivation to the medial prefrontal and anterior cingulate cortices, key regions in processing reward information and in goal-directed behavior (Knutson *et al*, 2001, 2003; Volz *et al*, 2005; Kringelbach and Berridge, 2009; Haber and Knutson, 2010), which are implicated in the pathophysiology of both depression and schizophrenia (Fornito *et al*, 2008; Drevets, 2001).

Taken together our results indicate an important role for the medial prefrontal cortex and anterior cingulate in the genesis of anhedonic symptoms (especially in schizophrenia). We show that the activity in these regions is reduced in schizophrenia compared with controls during unexpected reward receipt, that the degree of reward receipt related activation in this area is associated with the degree of motivation to continue playing a game, and that the degree of motivation to continue playing in our experiment relates to severity of anhedonia in schizophrenia.

### Limitations

One significant limitation of the current study is that all the schizophrenia patients and about half the depression patients were taking psychotropic medication, given that both of these classes of medication have been shown to affect brain activity during reward processing experiments. In experiments in healthy volunteers, certain antipsychotics and antidepressants have been shown to result in reduced reward-related brain activation (Pessiglione *et al*, 2006; Abler *et al*, 2007; Graf *et al*, 2014; Macovaneau, 2014); other experiments in healthy controls and in patients with schizophrenia have suggested that atypical antipsychotic medications may improve reward-related brain activation relative to placebo (Jocham *et al*, 2011) or to typical antipsychotic medication (Kirsch *et al*, 2007). The balance of the (limited) evidence suggests that antidepressant and (atypical) antipsychotic treatment help normalize brain responses to reward-related stimuli in patients with depression (Stoy *et al*, 2012) and schizophrenia (Nielsen *et al*, 2012), respectively. All of our schizophrenia participants were taking atypical antipsychotic medication. Given that we did not find significant differences between medicated *vs* unmedicated depressed participants and no associations between brain activity and dose of medication, it is not likely that medication completely explains the reward processing group differences observed in our study, though we recognize that studies in patients not taking medication are required to confirm our results. We note that there have been some previous studies documenting altered striatal reward prediction error signaling in unmedicated schizophrenia patients (eg, Schlagenhauf *et al*, 2014). All of our patient participants subjectively endorsed at least some degree of anhedonia, so our results may not be representative of patients with schizophrenia and depression who are not anhedonic. The measure of anhedonia that we used, the SHAPS, provides a summary measure of anhedonia and does not distinguish between different components.

The task we used delivered unpredictable rewards, and as such rewards were surprising (and hence associated with positive reward prediction error). The activity formed by our contrast of interest (unexpected reward receipt *vs* full miss), could reflect either activity due to reward value or reward prediction error, hence inferences should be drawn accordingly.

Our findings of some similarities in dysfunction of the brain representations of unexpected reward receipt in depression and schizophrenia is broadly consistent with recent reports that examined a related area of reward processing—reward anticipation—in patients with a variety of psychiatric disorders (Hägele *et al*, 2014; Arrondo *et al*, 2015). In a fascinating analysis, Hägele *et al* (2014) pooled a large number of patients with depression, schizophrenia, ADHD, mania, and alcohol dependence, who had all previously taken part in separate case-control studies using a reward anticipation task, and found evidence of right ventral striatal dysfunction linked to depressive symptom severity across diagnostic categories. This finding, and our results indicating shared areas of reward-related pathophysiology across disorders, are highly relevant to the RDoC initiative, and are supportive of its dimensional approach to psychopathology. RDoC aims to increase research that validates new cross-diagnostic dimensions that will ultimately inform future diagnostic systems (Cuthbert, 2014). One proposed RDoc domain is ‘positive valence systems', and proposed dimensions within that domain include responsiveness to reward, approach motivation, reward prediction error, and reward learning. Our approach is in keeping with the RDoc framework, and our results may encourage further research examining areas of commonality (and difference) across various dimensions of reward processing in a cross-diagnostic fashion.

## Conclusion

In this study we provide evidence of similar hypofunction of the striatum and orbitofrontal cortex in both schizophrenia and depression during receipt of an unexpected reward. Similarities in the way that reward processing was disturbed in both disorders suggests that there may be at least some shared pathophysiology common to both disorders, which may indicate that similar treatments could be effective in both conditions in some circumstances.

We also show that the degree of frontal activation whilst playing a computer reward game is linked to subjective experience of motivation and that measure in turn is associated with severity of anhedonia (in schizophrenia), suggesting a possible causal pathway between brain activity, immediate measures of motivation, and longer-term levels of anhedonia.

## Funding and disclosure

Dr Robbins has received research support from or served as a consultant to Cambridge Cognition, Eli Lilly, GlaxoSmithKline, and Lundbeck. Dr Clark has served as a consultant to Cambridge Cognition; the Centre for Gambling Research at UBC was funded by the Province of BC government and the British Columbia Lottery Corporation. Dr Ziauddeen has been jointly funded by the Wellcome Trust and GlaxoSmithKline on the Translational Medicine and Therapeutics programme. Professor Fletcher has received funds from GlaxoSmithKline for consultation services and from Astra Zeneca for a lecture. Drs Segarra, Metastasio, Spencer, Dudas, Arrondo, and Murray and Mr Reinders have no financial interests to disclose.

## Figures and Tables

**Table 1:** Subjects' Demographics and Clinical Characteristics

	**Controls (*n*=21)** **mean/SD**	**Depression (*n*=24)** **mean/SD**	**Schizophrenia (*n*=21)** **mean/SD**	**F/X2**	***P*-value**	***Post hoc* tests**
Age	34.33/±10.11	33.08±9.15	32.24±7.44	0.29	0.749	
Gender (male/female)	17/4	17/7	18/3	1.712	0.425	
Handedness (right/left)	18/3	22/2	16/5	2.004	0.367	
White-British	17	20	17	0.044	0.978	
Culture fair (IQ)	114.24±19.97	107.08±16.6	100.20±19.28	2.93	0.061	
Education (years)	14.85±1.93	13.43±2.21	13.50±2.09	3.01	0.057	
BPRS			43.14±11.89			
BDI total	4.29±4.89	32.62±7.06	21.05±8.95	88.19	<0.001	C<D C<S S<D
SHAPS	23.38±3.58	33.42±6.81	29.19±6.2	17.00	<0.001	C<D C<S S<D
Antipsychotics (chlorpromazine equivalents)		a	377±424			
Antidepressants prescribed		13 (54%)	8 (38%)			

a Four depression participants were prescribed low-dose antipsychotic medication; see Supplementary Text for further medication details.

Any significant *post hoc* test results for two-group comparisons (*p*<0.05) are also indicated by the use of ‘greater than' symbols.

**Table 2:** Unexpected Reward Receipt Group Differences (One-Way ANOVA Implemented in FSL, FWE Corrected, Cluster Threshold *Z*>2.0, *p*<0.05.)

**Anatomical location**	**Cluster size**	***Z*-score**	**MNI coordinates**			***Post hoc*** **test**
			***x***	***y***	***z***	
Medial frontal cortex (right superior frontal gyrus and paracingulate)	754	3.52	14	30	56	C>S; C>D; D>S
Right ventral striatum orbitofrontal cortex, thalamus, and midbrain	1977	4.25	8	6	−2	C>D; C>S
Left lingual gyrus, occipital lobe	1577	3.72	−6	−80	2	C>D; C>S
Left orbitofrontal cortex	1120	3.4	−48	40	−22	C>D; C>S
Right inferior and middle temporal gyri	742	3.52	62	−26	−22	C>D; C>S
Right occipital pole and right cerebellum	2631	4.85	−20	−100	6	C>S
Posterior cingulate gyrus	830	4.33	0	−24	30	C>S
Left cerebellum	705	4.23	−14	−76	−34	C>S
Right angular and supramarginal gyri, parietal lobe	1815	4.75	54	−54	40	C>S; D>S
Left angular and supramarginal gyri, parietal lobe	1016	4.02	−40	−62	40	C>S; D>S

Abbreviations: C, controls; D, depression; MNI, Montreal Institute of Neurology; S, schizophrenia.

The *Z*-score for the maximum peak value of each cluster is provided. Any significant *post hoc* test results for two-group comparisons (*p*<0.05) on extracted contrast parameter estimates are also indicated for each cluster by the use of ‘greater than' symbols.
