# Supplementary material for: Abnormal Frontostriatal Activity During Unexpected Reward Receipt in Depression and Schizophrenia: Relationship to Anhedonia
Source: Neuropsychopharmacology. 2016 Jan 20;41(8):2001–10. doi: 10.1038/npp.2015.370 (PMC4820052; doi:10.1038/npp.2015.370)
Supplement: Supplementary Text [file npp2015370x1.doc]

**Supplementary text: Abnormal frontostriatal activity during unexpected reward receipt in depression and schizophrenia: relationship to anhedonia.**

***Nuria Segarra PhD, Antonio Metastasio MD, Hisham Ziauddeen MD PhD, Jenny Spencer MRCPsych, Niels R Reinders Msc, Robert B Dudas PhD, Gonzalo Arrondo PhD, Trevor W Robbins PhD FRS, Luke Clark PhD, Paul C Fletcher PhD FMedSci, Graham K Murray MD PhD.***

**Methods:**

*MRI methods*: A Siemens Trio Tim operating at 3 T was used to collect imaging data. Gradient-echo T2*-weighted echo planar images depicting BOLD contrast were acquired from 32 non-contiguous oblique axial planes to minimize signal drop-out in ventral regions. TR = 2s; echo time = 30ms; flip angle = 78; voxel size = 3.14 x 3.14 x 3.75mm^3^, matrix size 64x64; bandwidth 2232 HZ/Px. A high-resolution T1-weighted three-dimensional MP-RAGE structural image was also acquired for use in spatial normalization of the EPI series. Imaging data was analysed using FSL software (FMRIB's Software Library, [www.fmrib.ox.ac.uk/fsl](http://www.fmrib.ox.ac.uk/fsl)). Individual participants’ data were analysed using FEAT v5.98 (FMRI Expert Analysis Tool). EPI images were realigned and motion-corrected; non-brain components were removed from the images. Functional timeseries were spatially smoothed with a Gaussian kernel (6 mm, full-width half-maximum), high-pass filtered (130 s cut off), and the images were registered first to a whole brain echo-planar image, then to an anatomical scan obtained from the corresponding subject, and normalised to a standard template (MNI, voxel size 2x2x2mm). The first 6 volumes were discarded to allow for T1 equilibration effects. Each 60 trial EPI run lasted a maximum of 630 repetitions (21 minutes), but was terminated early on 60 trials completion.

Our use of a cluster correction with an initial cluster threshold of z=2, and inference corrected for multiple comparison at p=0.05, necessitates a cluster of size of over 250 voxels according to Gaussian random field theory (Worsley 2003). Given that a key region of interest, the ventral striatum, contains a total of 1600 voxels, this is reasonable, although it would not detect small areas of focal difference.

*Participants*: Patient participants subjectively endorsed a degree of anhedonia: they answered yes when asked in a telephone or face to face study screening interview whether they had any difficulties enjoying themselves or had a loss of interest in things they used to enjoy, confirmed in the actual experiment using the BDI, SHAPS and also anhedonia items on the SANS. Denial of problems with interest and enjoyment was an exclusion.

Demographics and all other non-imaging statistical tests were compared across groups using 1-way ANOVAS or Kruskal-Wallis tests and post-hoc uncorrected t-tests. All behavioural analyses were carried out using SPSS v21 (Armonk, NY, USA) and results were considered statistically significant when p-value<0.05.

**Results:**

All patients with schizophrenia were taking atypical antipsychotic medication (specifically clozapine (n=11), aripiprazole (n=3), risperidone (n=1), olanzapine (n=2), clozapine and aripirazole (n=1), clozapine and quetiapine (n=1)); two patients were taking a combination of typical and atypical medication: one patient was taking a combination of clozapine and the typical antipsychotic medication sulpiride, and one patient was taking a combination of risperidone and the typical antipsychotic medication flupenthixol. The mean chlorpromazine equivalent dose was 377 (standard deviation 424) mg/day (Kroken et al, 2009). Eight patients with schizophrenia were additionally taking antidepressant medication: citalopram (n=4) 20-40 mg, fluoxetine (n=1) 20 mg, mirtazapine (n=1) 45 mg, venlafaxine (n=2) 150-225mg. Four depression patients we taking low dose antipsychotic medication in addition to an antidepressant (2 risperidone, 2 quetiapine; the mean chlorpromazine equivalent dose for these four was 150 mg/day). Thirteen depression patients were taking antidepressant medication (venlafaxine 75mg-225mg (n=5), mirtazapine (30mg-45mg) (n=2), fluoxetine 20mg (n=1), citalopram (30mg-60mg) (n=4); one took lofepramine 70mg and mirtazapine 45mg) and eleven were not taking any psychiatric medication. There was no significant difference in the parameter estimates in any of the clusters between medicated depression versus non-medicated depression participants and no significant correlation with dose of antipsychotic medication (chlorpromazine equivalents) in the schizophrenia group. We checked whether, within the schizophrenia group, the relationship between brain activation during the receipt of unexpected reward and the motivation to continue playing the game was confounded by dose of medication: the relationship remained significant after controlling for antipsychotic dose.

*Behavioural results, task wins:* the task was programmed so that if a participant answered every trial in time, he/she would win on 20 occasions. However, being too late could result in a few missed wins. The mean number of wins for each group was 19.4 in control (95% confidence intervals 19.1-19.8), 19.3 (18.7-19.8) in depression and 18.7 (18.0-19.4) in schizophrenia. The number of missed wins due to too-late responses for each group was 0.57 (0.20-0.94) in controls, 0.75 (0.25-1.25) in depression and 1.33 (0.63-2.02) in schizophrenia (F=2.3, df=2, 64, p=0.11).

*Behavioural results, relationship between task measures and SHAPS anhedonia:* as both task behavioural measures (“When the second picture matched the chosen picture you won money. How much did you like the feeling of winning money?” and, “When the second picture matched the chosen picture you won money. Did this make you want to play more?”) were highly correlated, we examined their relation with SHAPS anhedonia in a stepwise regression model with SHAPS as the outcome variable and the task measures as predictors (whole sample; stepping entry criteria was F probability 0.05 and removal F probability 0.1). The task ‘liking’ measure was eliminated and the motivation measure remained in the model.

*MRI results, two-sample T-tests:* in the main text we report the results of ANOVA analyses to find differences between the three groups and our inferences are based on those and associated post-hoc tests. As suggested by an anonymous referee we here report the results of two group comparisons: we show voxelwise uncorrected results in Supplementary Figures 5,6,7,8.

We also conduct further posthoc T-tests within regions defined by the group F-test, here implemented within FSL at the voxel level (as opposed to our previous post-hoc tests implemented in SPSS). These data are reported using family wise error cluster correction p<0.05; in order to provide increased anatomical specificity (requested by a referee), in these posthoc tests we raise the initial Z cluster forming threshold to z=2.3 which has the effect of favouring the detection of relatively smaller clusters compared to a lower z. See Supplementary Figures S9 and S10.

*MRI results using exclusive masking:* following the suggestion of an anonymous referee, we show results of each patient group versus controls (voxelwise uncorrected p<0.005), exclusively masked by results of the other patient group versus controls (using a fairly liberal exclusive mask threshold of p<0.05 to help ensure specificity). By using exclusive masking we ensure than when looking for regions that differentiate, say, schizophrenia from controls, we mask out any area in which there is reasonable suspicion that there could be depression versus controls differences. In this way, we depict which areas are specifically abnormal in each group compared to controls. The results of these group comparisons with exclusive masking demonstrate more considerably more widespread specific schizophrenia versus controls differences than specific depression versus controls differences (Supplementary Figures 11,12). When examining supplementary figures 5,6,9,10, 11 and 12, it can be seen that some areas demonstrate more widespread abnormalities in schizophrenia than in depression, such as parts of the basal ganglia, lateral frontal cortex, lateral parietal cortex (angular gyri and supramarginal gyri) and cerebellum (the lateral parietal lobe and cerebellum also came through as abnormal in schizophrenia but not depression in the cluster-corrected analysis presented in the main text). We note that the exclusively masked analyses are not corrected for multiple comparisons.

*MRI results, interaction with personal control and near-miss effect:* on the suggestion of an anonymous referee, we ran a new analysis and checked whether the response to a reward (versus a full miss) differed according to whether the participant had selected the play icon or the computer had. There was no effect in controls or schizophrenia (z=2, p<0.05 Family wise error corrected). There was one cluster in depression at this threshold with its peak voxel in the occipital cortex, but also extending to the cerebellum (9300 voxels, peak z statistic=3.7, peak voxel MNI co-ordinates x=26, y=-90, z=-8). Regarding the near miss effect (near miss versus full miss), there was a significant group difference in the occipital cortex (x=20,y=-92,z=6; voxel number =1001, peak z-statistic=3.04); here the schizophrenia showed reduced activation compared to the other two groups (p=0.04 versus controls, p=0.03 versus depression). Analyses of personal control and near-miss effect may have been limited in sensitivity given the fact that we ran two runs of the task in this psychiatric study as opposed to a three-run version used previously in healthy volunteers.

References:

Kroken RA, Johnsen E, Ruud T, Wentzel-Larsen T, Jørgensen HA (2009). Treatment of schizophrenia with antipsychotics in Norwegian emergency wards, a cross-sectional national study. BMC Psychiatry. 2009 May 16;9:24.

Worsley K (2003), “Statistical analysis of activation images” in Functional Magnetic Resonance Imaging: An Introduction to Methods. Edited by Jezzard, P., Matthews, P.M., and Smith. S. Oxford University Press. Oxford.
