# Supplementary material for: Abnormal Frontostriatal Activity During Unexpected Reward Receipt in Depression and Schizophrenia: Relationship to Anhedonia
Source: Neuropsychopharmacology. 2016 Jan 20;41(8):2001–10. doi: 10.1038/npp.2015.370 (PMC4820052; doi:10.1038/npp.2015.370)
Supplement: Supplementary Figure S1–S25 [file npp2015370x2.doc]

**SUPPLEMENTARY FIGURES**


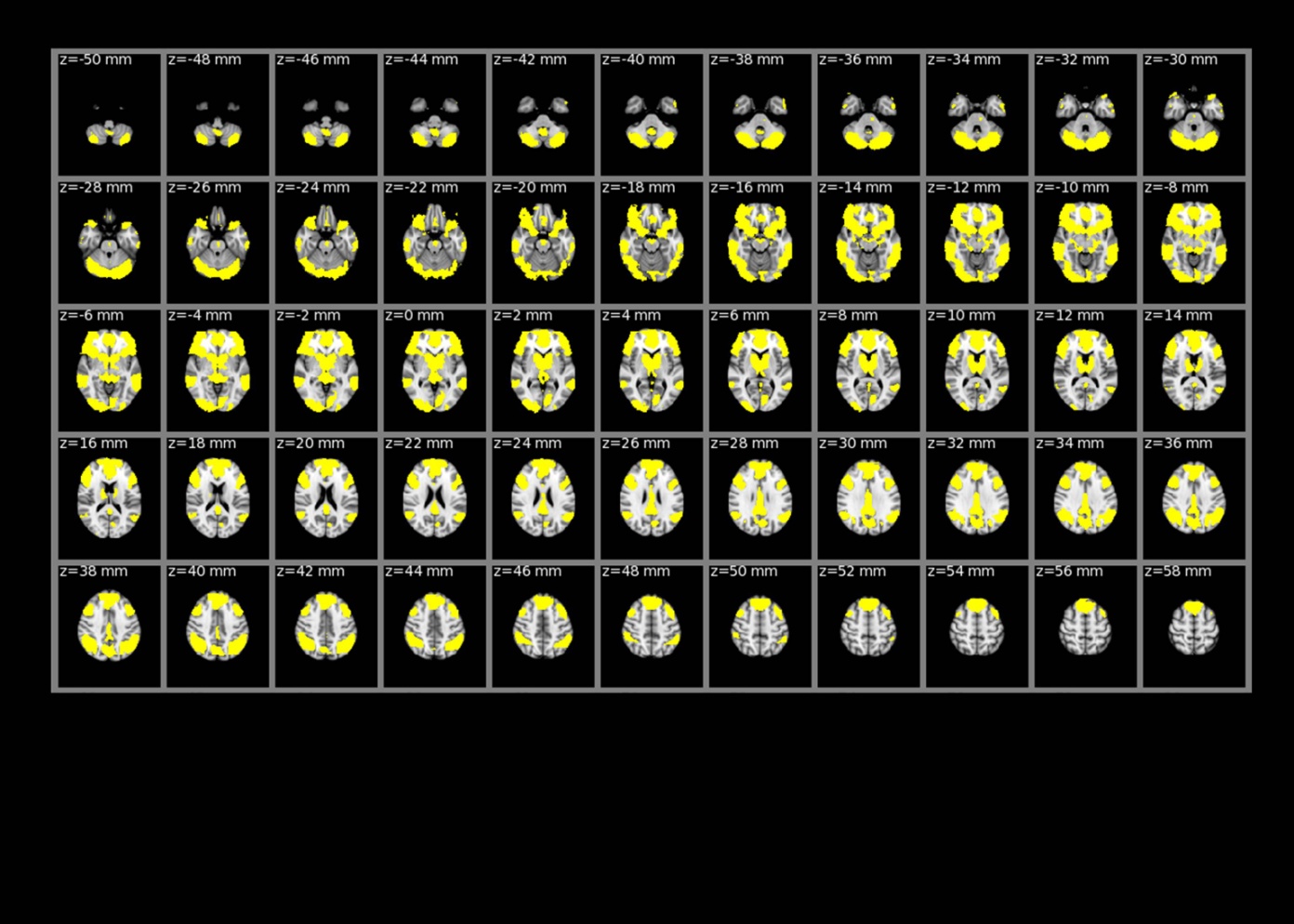


Supplementary Figure S1. fMRI results: receipt of unexpected reward: entire sample pooled analysis. The yellow colour indicates significant voxels thresholded at p<0.05 FWE corrected for illustrative purposes. Left hemisphere is shown in the right side of the image. Coordinates are expressed in mm, and in standard space.


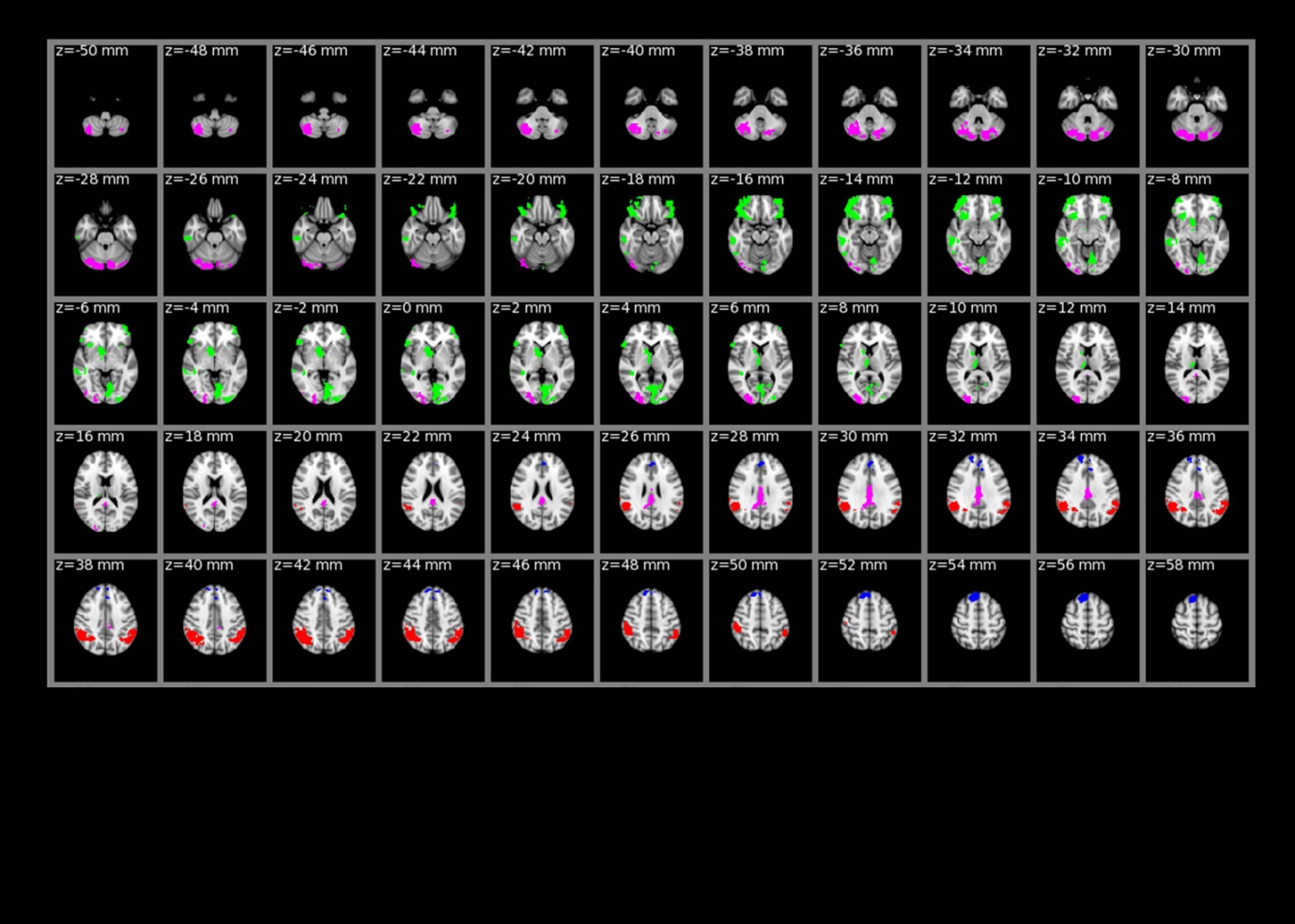


Supplementary Figure S2. fMRI results: receipt of unexpected reward. Between groups analysis using ANOVA. Coloured clusters indicate those clusters indicating significant group differences (cluster threshold Z>2.0 p<0.05 FWE whole brain corrected) and the particular colour indicates the results of post-hoc tests. Green areas are clusters where the Control group has greater activation compared to the Depression group and compared to the Schizophrenia group; Blue areas are clusters where Controls have greater activation compared to the Depression group and compared to the Schizophrenia group, and the Depression group has greater activation than the Schizophrenia group; Red areas are clusters where Controls have more activation than the Schizophrenia group and the Depression group has more activation than the Schizophrenia group; Lilac areas are clusters those where the Control group has more activation than the Schizophrenia group. Left hemisphere is shown in the right side of the image. Coordinates are expressed in mm, and in standard space.


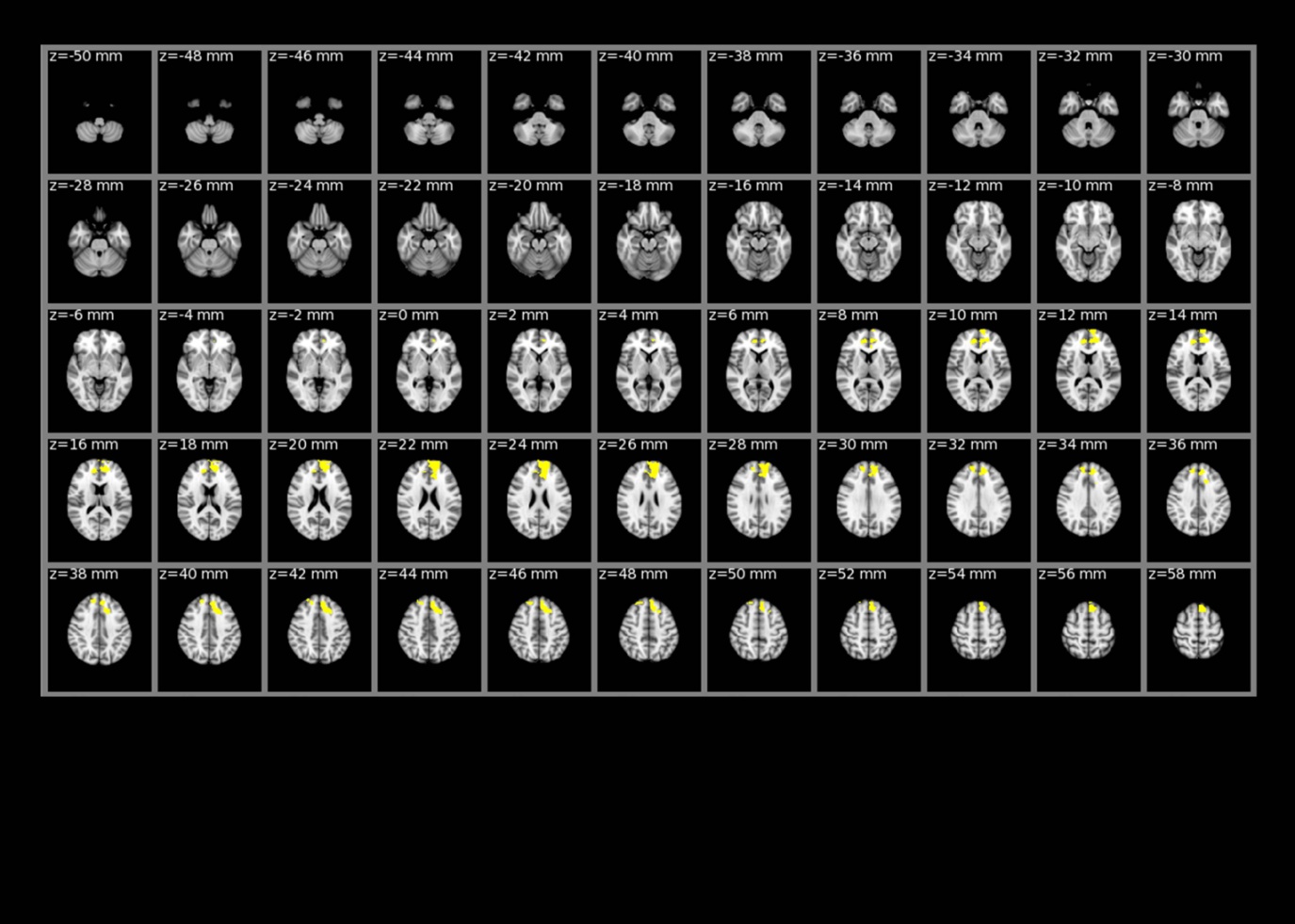


Supplementary Figure S3. fMRI results of linear regression of brain activation during an unexpected win against subjective ratings of motivation (all subjects pooled, adjusted by group). Left hemisphere is shown in the right side of the image. Coordinates are expressed in mm, and in standard space. Yellow indicates a significant cluster, FWE whole brain corrected, cluster threshold Z>2.0 p<0.05.


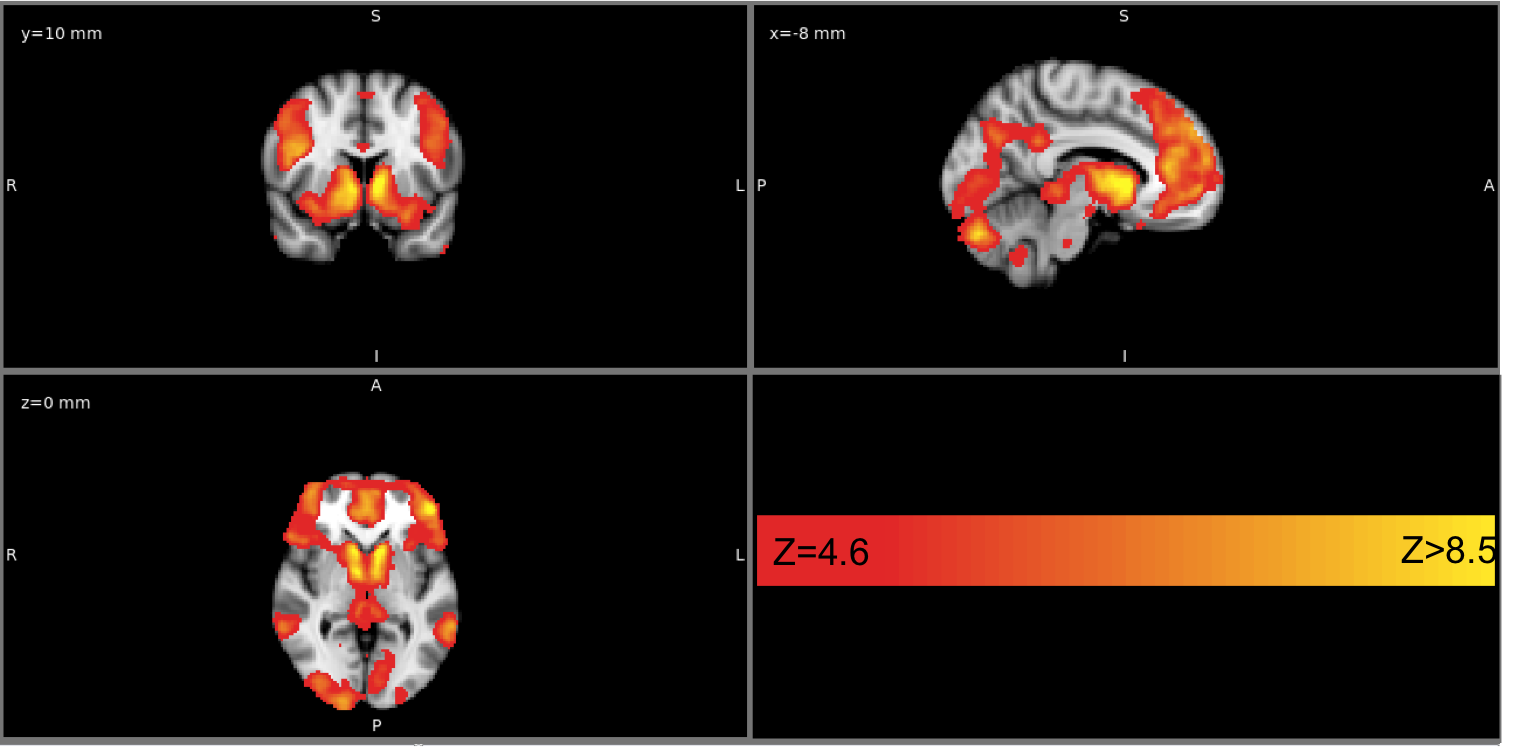


Supplementary Figure S4. fMRI results: receipt of unexpected reward: entire sample pooled analysis, thresholded at p<0.05 FWE corrected for illustrative purposes, with the highest z statistic values being shown in yellow (see colour bar in bottom right panel). Coordinates are expressed in mm, and in standard space. L, left; R, right; S, superior; I, inferior; A, anterior; P, posterior.


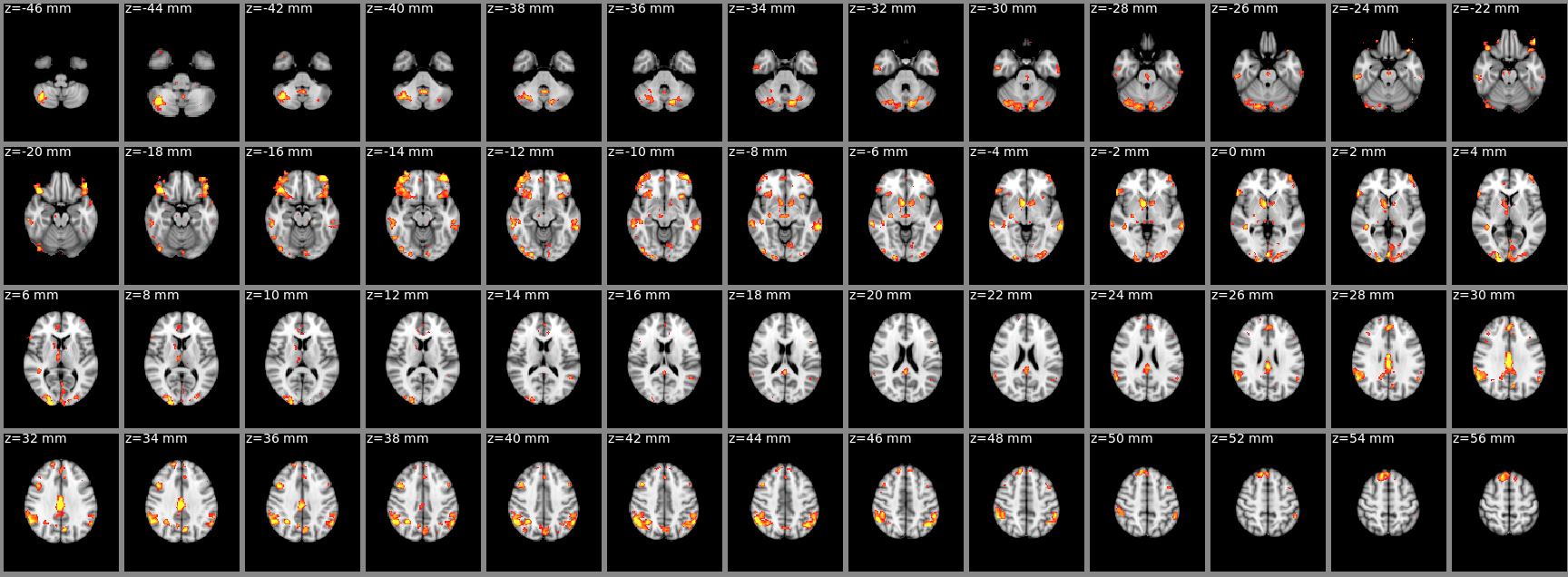


Supplementary Figure S5. Results of two-group comparison T-Tests (receipt of unexpected reward): The coloured areas are voxels where activation in controls > in schizophrenia. P<0.005 (voxelwise analysis, uncorrected for multiple comparison). Here, yellow voxels have z-score of 3.5 or greater. Left hemisphere is shown in the right side of the image. Coordinates are expressed in mm, and in standard space.


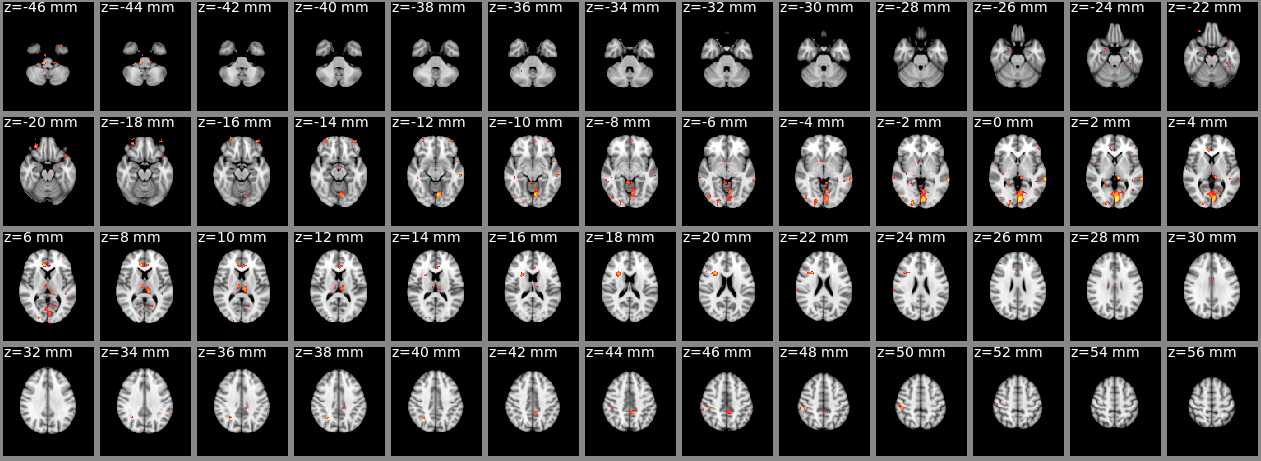


Supplementary Figure S6. Results of two-group comparison T-Tests (receipt of unexpected reward): The coloured areas are voxels where activation in controls > in depression. P<0.005 (voxelwise analysis, uncorrected for multiple comparison). Here, yellow voxels have z-score of 3.5 or greater. Left hemisphere is shown in the right side of the image. Coordinates are expressed in mm, and in standard space.


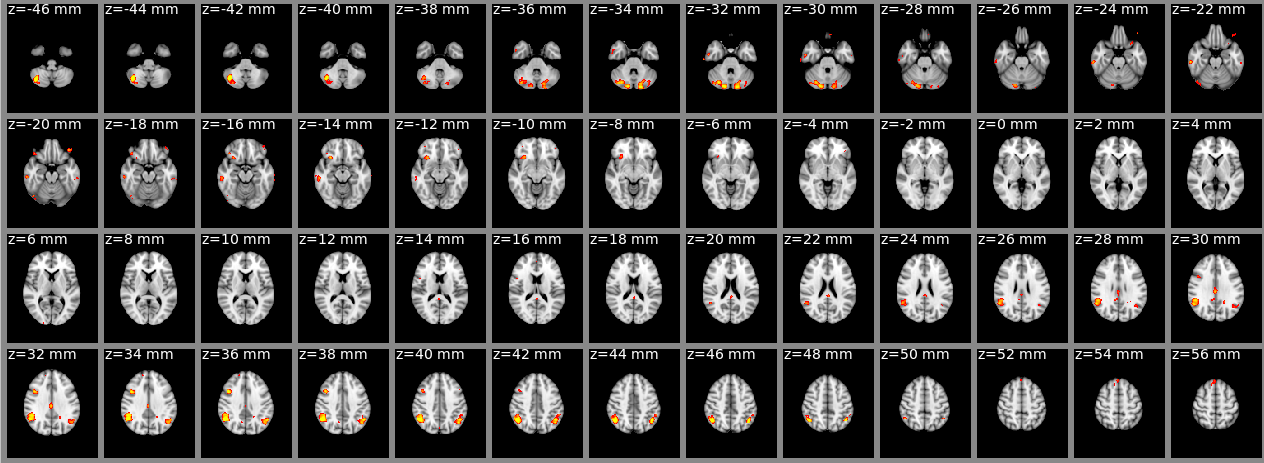


Supplementary Figure S7. Results of two-group comparison T-Tests (receipt of unexpected reward): The coloured areas are voxels where activation in depression > in schizophrenia. P<0.005 (voxelwise analysis, uncorrected for multiple comparison). Here, yellow voxels have z-score of 3.5 or greater. Left hemisphere is shown in the right side of the image. Coordinates are expressed in mm, and in standard space.


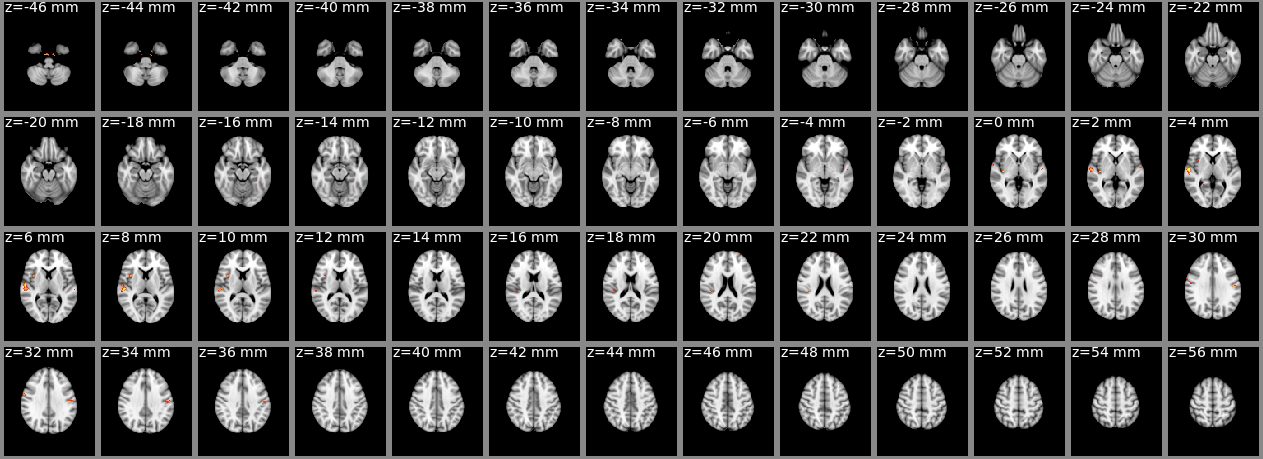


Supplementary Figure S8. Results of two-group comparison T-Tests (receipt of unexpected reward): The coloured areas are voxels where activation in schizophrenia > in depression. P<0.005 (voxelwise analysis, uncorrected for multiple comparison). Here, yellow voxels have z-score of 3.5 or greater. Left hemisphere is shown in the right side of the image. Coordinates are expressed in mm, and in standard space.


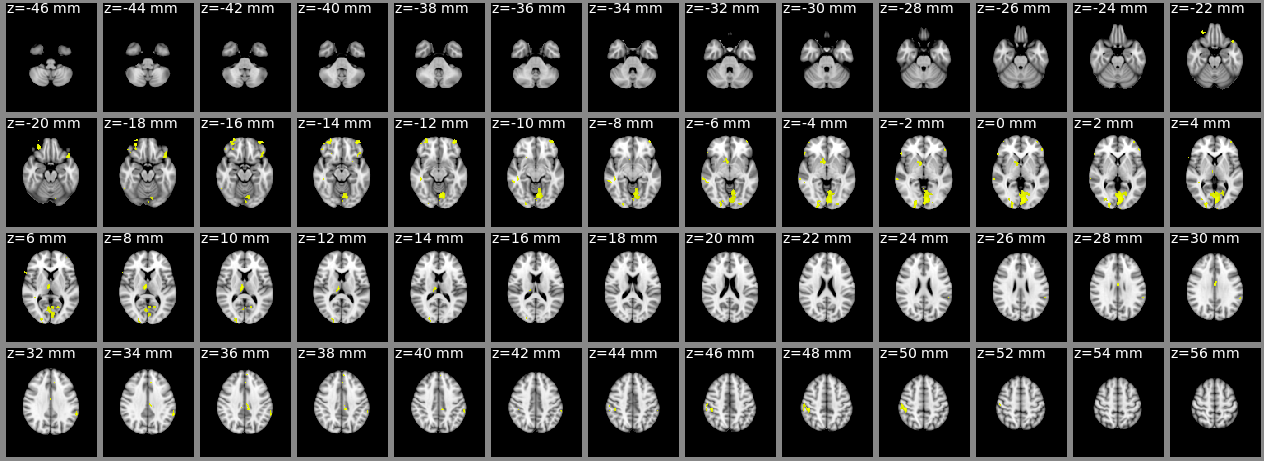


Supplementary Figure S9. Results of post-hoc two-group comparison T-Tests (receipt of unexpected reward): The yellow areas are clusters (initial cluster threshold z=2.3) where controls have greater activation than depression p<0.05 family wise error cluster corrected across the ROIs defined by the three group F test. Left hemisphere is shown in the right side of the image. Coordinates are expressed in mm, and in standard space.


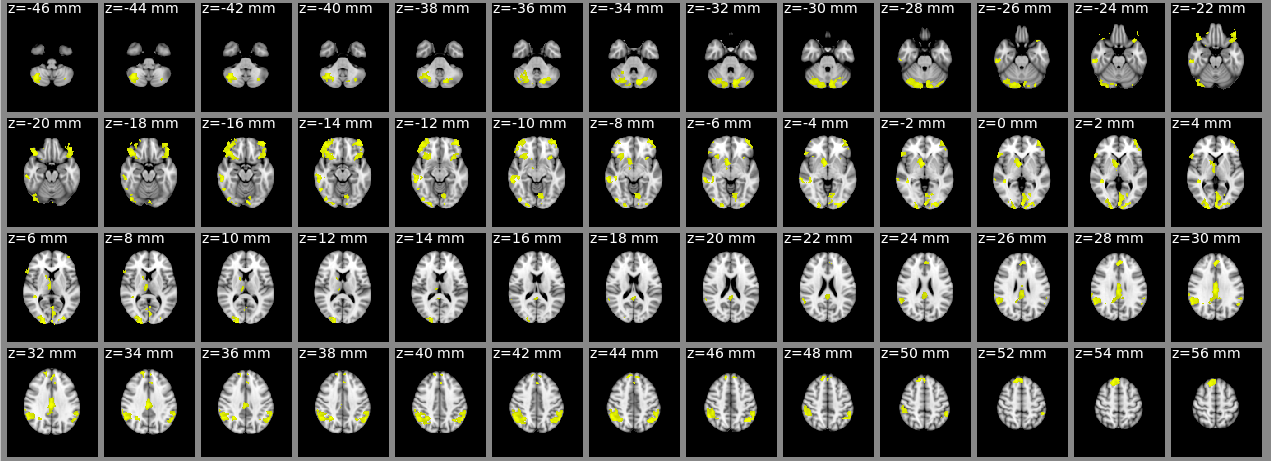


Supplementary Figure S10. Results of post-hoc two-group comparison T-Tests (receipt of unexpected reward): The yellow areas are clusters (initial cluster threshold z=2.3) where controls have greater activation than schizophrenia p<0.05 family wise error cluster corrected across the ROIs defined by the three group F test. Left hemisphere is shown in the right side of the image. Coordinates are expressed in mm, and in standard space.


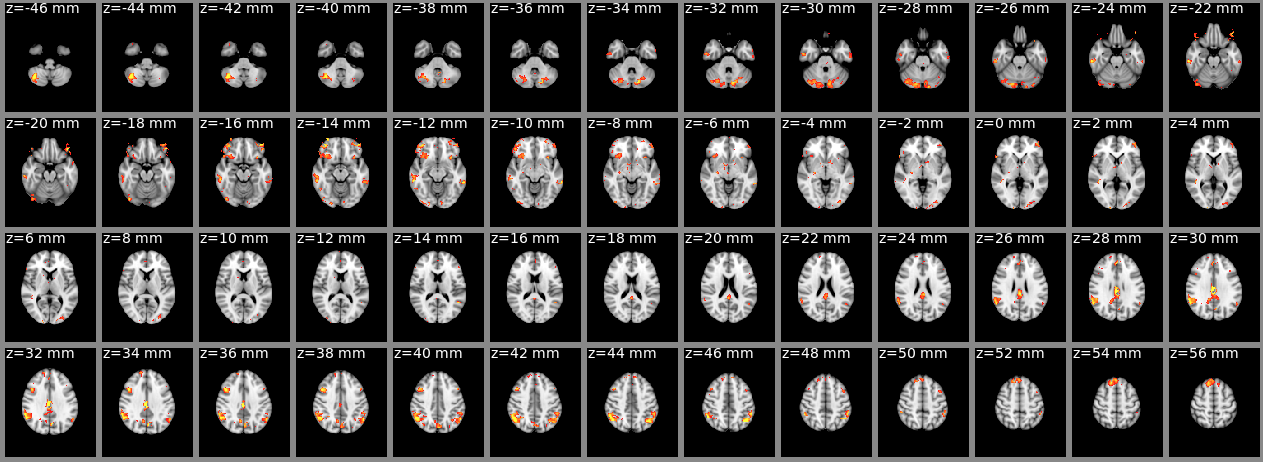


Supplementary Figure S11. fMRI results, unexpected reward receipt. Coloured voxels are those where activation in controls > in schizophrenia. P<0.005, uncorrected, exclusively masked by the results of controls > depression at p=0.05, uncorrected. Here, yellow voxels have z=3.5 or greater. This analysis depicts areas specifically abnormal in schizophrenia compared to controls. Left hemisphere is shown in the right side of the image. Coordinates are expressed in mm, and in standard space.


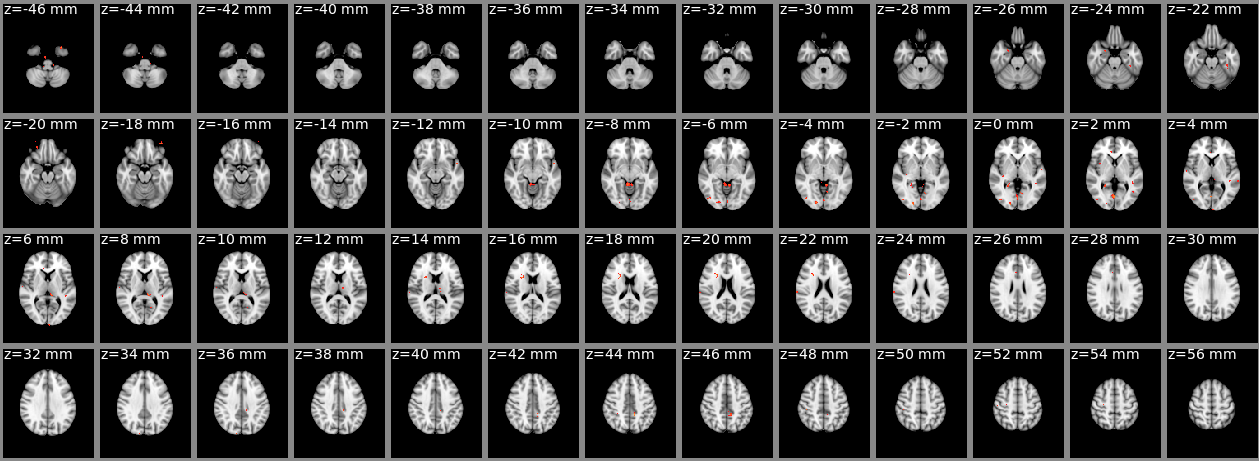


Supplementary Figure S12. fMRI results, unexpected reward receipt. Coloured voxels are those where activation in controls > in depression. P<0.005, uncorrected, exclusively masked by the results of controls > schizophrenia at p=0.05, uncorrected. Here, yellow voxels have z=3.5 or greater. This analysis depicts areas specifically abnormal in depression compared to controls. Left hemisphere is shown in the right side of the image. Coordinates are expressed in mm, and in standard space.


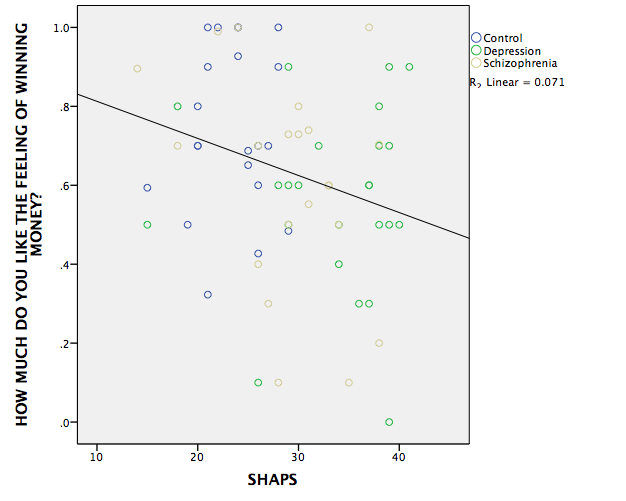


Supplementary Figure S13. Scatterplot of the task measure of “liking” versus SHAPS (p=0.03)


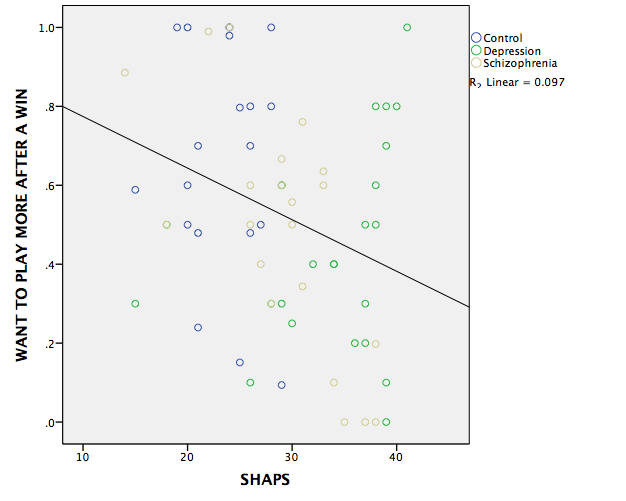


Supplementary Figure S14. Scatterplot of the task measure of “wanting” versus SHAPS (p=0.01)


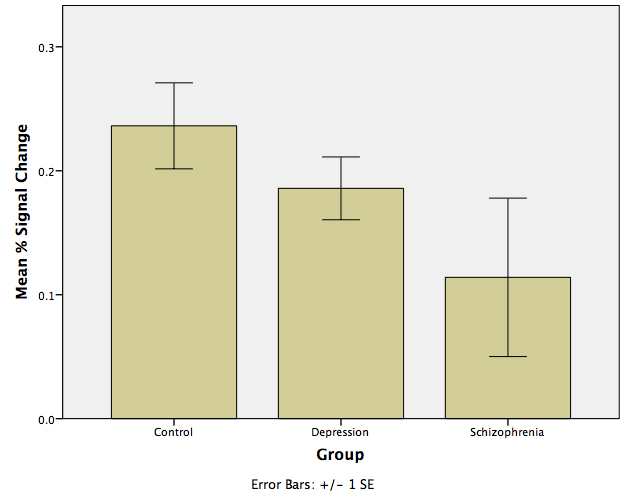


Supplementary Figure S15. % Signal change left cerebellar cluster (see Table 2)


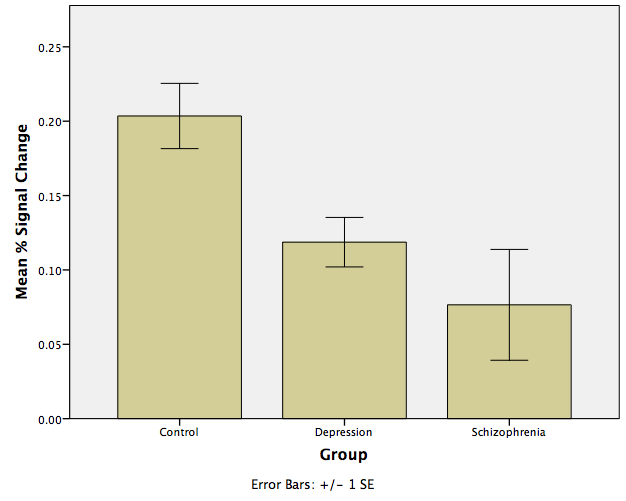


Supplementary Figure S16. % Signal change right inferior & middle temporal gyrus cluster (See Table 2)


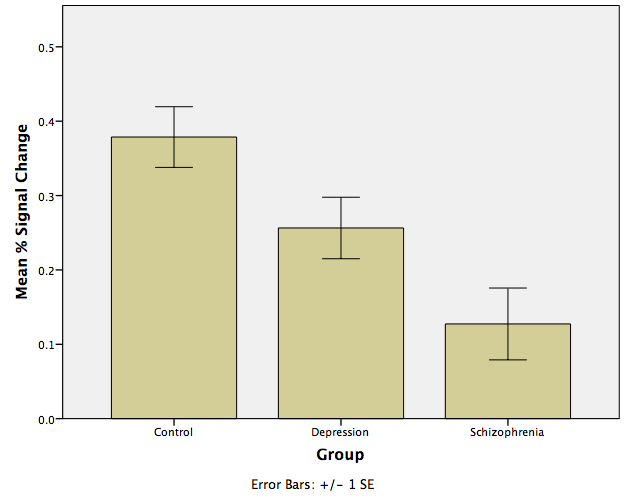


Supplementary Figure S17. % Signal change, medial frontal cluster. See Table 2.


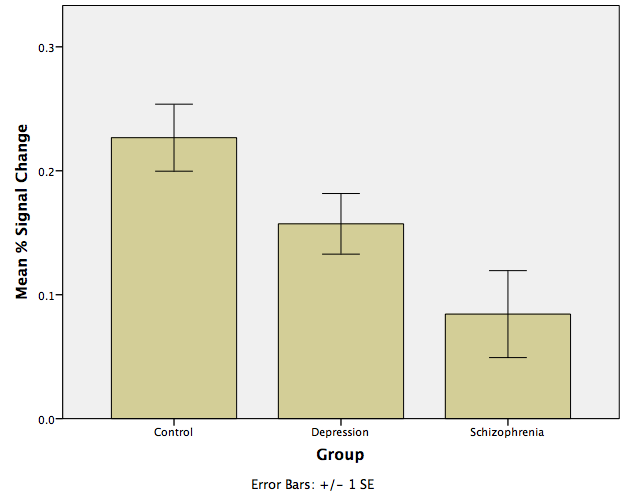


Supplementary Figure S18: % Signal change, posterior cingulate cluster (See Table 2)


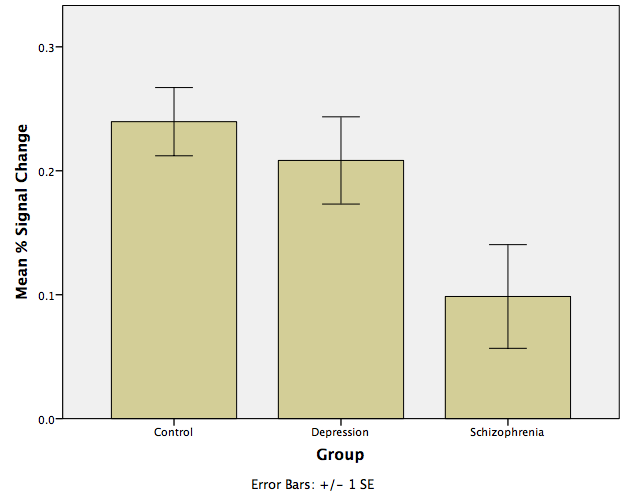


Supplementary Figure S19: % Signal change, left parietal lobe (angular/supramarginal gyrus) cluster (See Table 2)


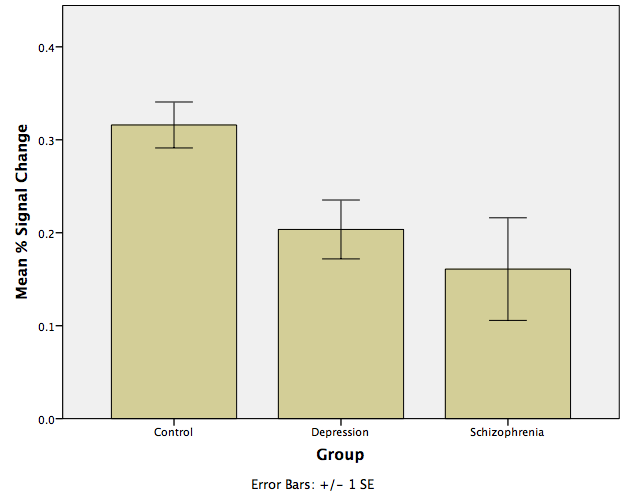


Supplementary Figure S20: % Signal change, left orbitofrontal cluster (See Table 2)


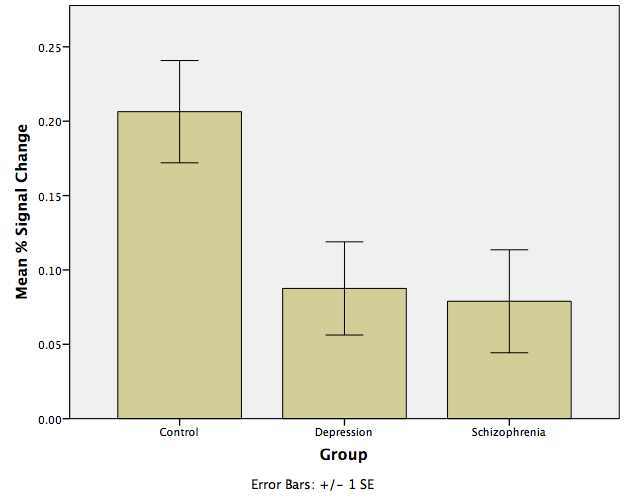


Supplementary Figure S21: % Signal change, left lingual gyrus cluster (See Table 2)


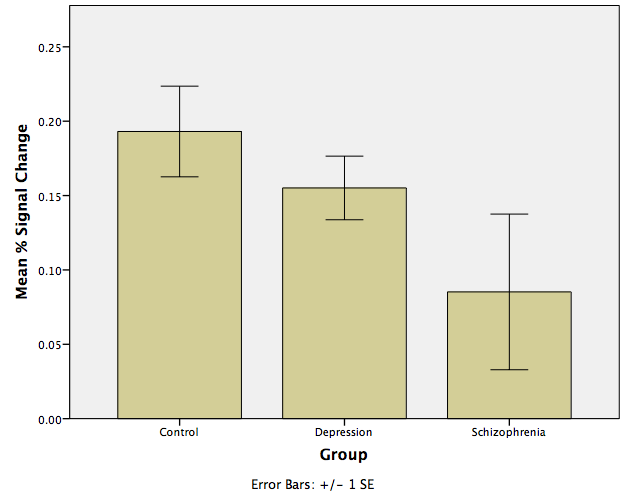


Supplementary Figure S22: % Signal change, right parietal lobe (angular/supramarginal gyrus) cluster (See Table 2)


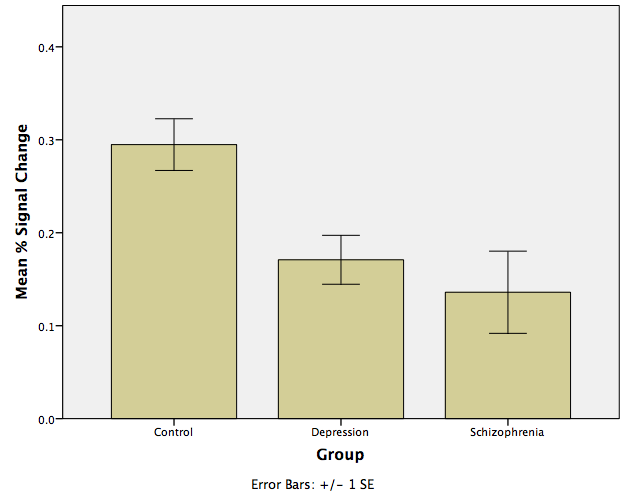


Supplementary Figure S23: % Signal change, right ventral striatal/orbitofrontal cluster (See Table 2)


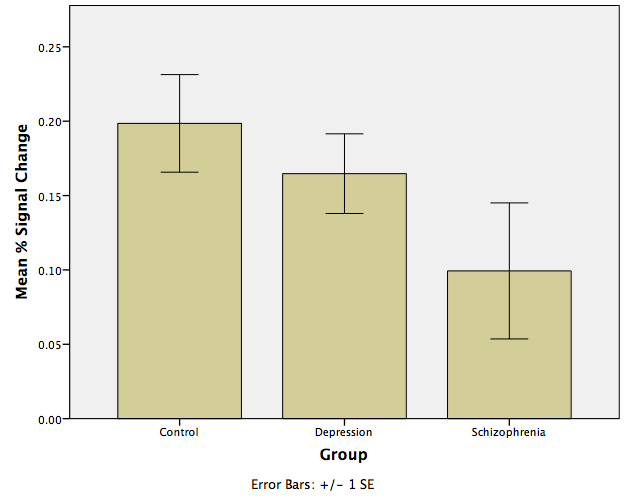


Supplementary Figure S24: % Signal change, right occipital pole/right cerebellum cluster (See Table 2)


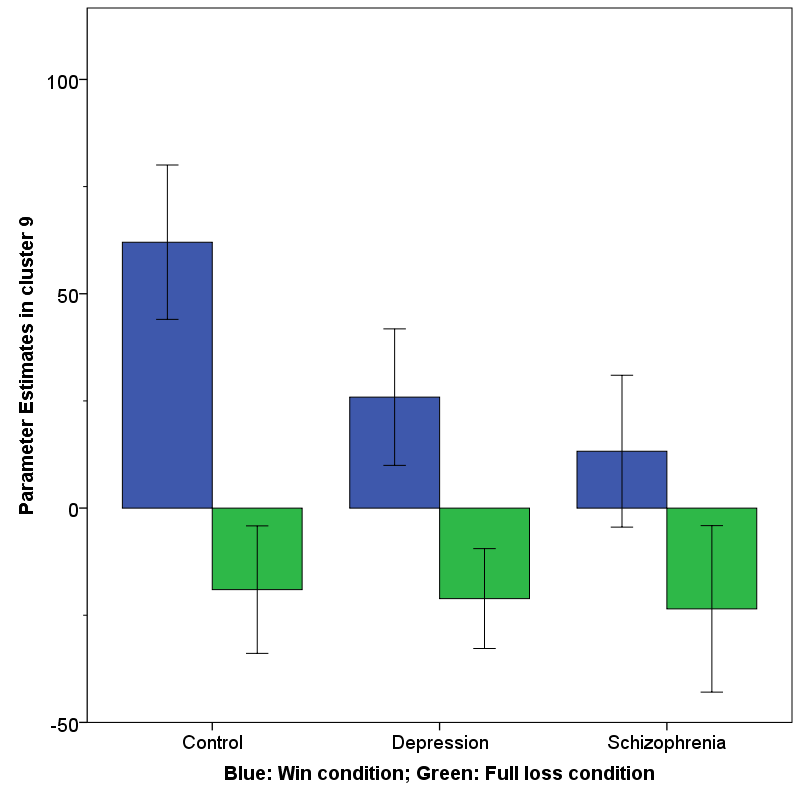


Supplementary Figure S25. Extracted parameter estimates from the right ventral striatal/orbitofrontal cortex cluster (Table 2, Figure 2) showing that group differences in the contrast of unexpected reward receipt versus full miss are driven by reduced brain response to unexpected reward. Error bars are 95% confidence intervals.
